# Placental transfer of tofacitinib in the *ex vivo* dual-side human placenta perfusion model

**DOI:** 10.1016/j.crtox.2024.100149

**Published:** 2024-01-08

**Authors:** Gaby A.M. Eliesen, Milou Fransen, Hedwig van Hove, Petra H.H. van den Broek, Rick Greupink

**Affiliations:** a Department of Pharmacology and Toxicology, Radboud University Medical Center, Nijmegen, the Netherlands; b Centre for Safety of Substances and Products, National Institute for Public Health and the Environment, Bilthoven, the Netherlands

**Keywords:** Cotyledon perfusion, Drug disposition, Drug safety, Reproductive toxicology, Fetal drug exposure, Pharmacokinetics, Tyrosine kinase inhibitors

## Abstract

Tofacitinib is a small molecule Janus kinase (JAK) inhibitor, introduced to the European market in 2017, for the treatment of rheumatoid arthritis, psoriatic arthritis and ulcerative colitis. In the treatment of women with autoimmune diseases, pregnancy is a relevant issue, as such diseases typically affect women in their reproductive years. Currently, there is limited data on the use of tofacitinib during pregnancy. To estimate the extent of placental transfer in the absence of clinical data, we conducted *ex vivo* dual-side perfused human placental cotyledon perfusions. Term placentas were perfused for 180 min with tofacitinib (100 nM, added to the maternal circuit) in a closed-closed configuration. At the end of the perfusions, drug concentrations in the maternal and fetal reservoirs were near equilibrium, at 35.6 ± 5.5 and 24.8 ± 4.7 nM, respectively. Transfer of tofacitinib was similar to that observed for the passive diffusion marker antipyrine (100 µg/mL, added to the maternal reservoir). Final antipyrine maternal and fetal concentrations amounted to 36.9 ± 3.0 and 36.7 ± 1.3 µg/mL, respectively. In conclusion, in the *ex vivo* perfused placenta tofacitinib traverses the placental barrier rapidly and extensively. This suggests that substantial fetal tofacitinib exposure will take place after maternal drug dosing.

## Highlights

• *Ex vivo* dual-side perfused human placental cotyledon perfusions were used to estimate placental transfer of tofacitinib.
• Tofacitinib traverses the placental barrier rapidly and extensively.
• Transfer of tofacitinib was similar to that of the passive diffusion marker antipyrine.
• This suggests that substantial fetal tofacitinib exposure will take place after maternal drug dosing.

## Graphical abstract

**Figure:**
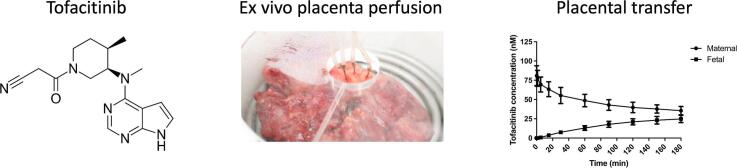

## Introduction

Tofacitinib (Xeljanz) is a small molecule Janus kinase (JAK) inhibitor, introduced to the market in Europe in 2017 for the treatment of rheumatoid arthritis, psoriatic arthritis and ulcerative colitis (European Medicines Agency, 2017). Tofacitinib inhibits all members of the JAK family, which are intracellular, non-receptor tyrosine kinases that transduce cytokine-mediated signals via the JAK-STAT pathway. Excessive JAK-STAT signaling is associated with immune disorders (Villarino et al., 2017), making tofacitinib a relevant therapy for the indications mentioned above. Tofacitinib is not a first line therapy, but it is indicated in patients with an insufficient response to conventional disease-modifying drugs (DMARDs) (European Medicines Agency, 2017).

In the treatment of women with autoimmune diseases, pregnancy is a relevant issue as such diseases typically affect women in their reproductive years. Generally speaking, pharmacotherapy during pregnancy is kept to a minimum, in order to minimize potential adverse effects on the fetus. However, for women living with auto-immune diseases who suffer from active disease episodes, it is advised to continue treatment during pregnancy in order to maintain disease remission. Active disease episodes are associated with adverse pregnancy outcomes. This is for example the case for ulcerative colitis (Gotestam Skorpen, 2016, McConnell and Mahadevan, 2016, de Jong and Dolhain, 2017, Cornish, 2007, Broms, 2014).

Currently, tofacitinib is contraindicated during pregnancy since it is a known teratogenic compound in rats and rabbits (European Medicines Agency, 2017). Still, in the context of phase II and phase III clinical trials, exposure to tofacitinib during the first trimester has been reported as women became unexpectedly pregnant while on tofacitinib treatment. Post marketing surveillance resulted in more cases of maternal tofacitinib exposure and pregnancy monitoring is still ongoing in a pregnancy registry (Clowse, 2016, Mahadevan, 2018). The available evidence from these studies has recently been summarized by Fernandez-Sanchez and others (Fernandez-Sanchez, 2021). A total of 120 cases of maternal exposure during the first trimester are reported. The outcomes were as follows: 48 healthy new-borns (40 %), 15 spontaneous abortions (12.5 %), two congenital malformations (1.7 %), 14 voluntary medical terminations (11.7 %), and 41 pending or lost to follow-up (34.1 %). No fetal or neonatal deaths were identified (Fernandez-Sanchez, 2021). Based on these outcomes, the percentages of spontaneous abortions and congenital malformations in these patients are similar to the general population where the rate of spontaneous abortions is usually estimated to be 10–15 % and the prevalence of congenital malformations about 3–5 % (Centers for Disease Control and Prevention, 2008, Anonymous, 2022)**.** These percentages are similar to, or somewhat lower than, prevalences in patients with autoimmune diseases using biologics (Centers for Disease Control and Prevention, 2008, Gerosa, 2018, Goralczyk, 2020, Weber-Schoendorfer, 2015, Anonymous, 2022). Of note is that tofacitinib treatment was discontinued upon discovery of pregnancy and that concomitant medication was given in a substantial number of cases. The same authors, also published another case report in which a patient with psoriatic arthritis gave birth to a healthy newborn after 2–3 weeks exposure to tofacitinib after conception (Fernandez-Sanchez, 2021).

To date, to our knowledge there are no case reports available where women were exposed to tofacitinib in either the second or third trimester. As a consequence, there is no information on the potential obstetric pharmacology of the drug in the later phases of pregnancy. However, mechanistic *in vitro* and *ex vivo* studies with human tissues can provide some insight in what may be expected.

As exposure to a drug drives its ultimate effects, it is important to consider the determinants of fetal drug exposure. The route of exposure differs between the first weeks of pregnancy and the later stages. In early pregnancy, diffusion of compounds across the uterine wall and across the different cell layers of which the developing embryo are constituted, is the main route of exposure. At the same time, invasion of interstitial extravillous trophoblasts into the uterine wall results in remodeling of uterine tissue and spiral arteries, establishing the maternoplacental blood flow. From pregnancy week 10–12 onwards, fetal villi that harbor branches of the umbilical blood vessels, protrude into intervillous spaces filled with maternal blood. From now on, the placental villi constitute the maternal-fetal interface and form the main site of transplacental transfer. In the second and third trimesters of pregnancy, fetal drug exposure is determined by maternal and fetal placental blood flow and the handling of pharmaceuticals by cells in the fetal villi, such as syncytiotrophoblast cells facing the intervillous spaces and endothelial cells lining the fetal blood vessels (Turco and Moffett, 2019, Pollheimer, 2018).

It is generally assumed that tofacitinib may cross the placental barrier as it is a small and rather lipophilic molecule, but placental transfer is determined by other physicochemical pharmacokinetic aspects as well. This is for example illustrated by earlier e*x vivo* placenta perfusion studies that report large variations among the placental transfer of several small molecule kinase inhibitors other than tofacitinib (Eliesen, 2017, Jovelet, 2015). Additionally, some types of drugs are extensively metabolized and/or effluxed by the placenta, whereas others are not. For example, when looking at corticosteroids, cortisol is extensively metabolized by the placenta and is thought to be actively transported back to the maternal circulation (Stirrat, 2018). Prednisolone follows a similar pattern, whereas betamethasone reaches the fetal circulation at much higher levels compared to prednisolone (Addison, 1991, Blanford and Murphy, 1977, Ballard et al., 1975).

As it is difficult to predict placental transfer of a compound *a priori*, the aim of this study is to investigate the rate and extent of placental transfer of tofacitinib in the *ex vivo* dual-side human placenta perfusion model. A better understanding of the placental transfer of this drug, and the potential fetal exposure resulting from this, further aides in deciding whether there may be a therapeutic use for tofacitinib during pregnancy.

## Methods

### Ethics statement

Placentas from uncomplicated pregnancies were obtained from consenting women who underwent elective caesarean sections or vaginal delivery. For this, approval of the institutional medical ethical committee was obtained (CMO Arnhem/Nijmegen File 2014–1397) in line with The Code of Ethics of the World Medical Association (Declaration of Helsinki).

### Ex vivo placenta perfusion experiments

*Ex vivo* placental perfusions were performed as reported previously by Eliesen et al, with some adjustments (Eliesen, 2017). Briefly, fetal perfusion solution consisted of Krebs-Henseleit buffer enriched with 11.1 mM glucose, 0.5 mL/l heparin 2500 IU, 34 g/l human albumin (Albuman) and 50 µg/mL FITC-dextran (40 kDa). Maternal perfusion solution consisted of Krebs-Henseleit buffer supplemented with 11.1 mM glucose, 0.5 mL/l heparin 2500 IU, 29 g/l human albumin (Albuman), 100 µg/mL antipyrine and tofacitinib (100 nM). Stock solutions of tofacitinib were prepared in DMSO and final DMSO concentrations in the perfusion buffer were < 0.5 %. Perfusion solutions were kept at 37 °C and the maternal buffer was oxygenated with 95 % O_2_/5% CO_2_ and the fetal buffer was gassed with 95 % N_2_/5% CO_2_ during the whole experiment to mimic physiological conditions.

Directly after delivery, a single intact cotyledon was selected and a fetal artery and associated vein were cannulated, establishing a fetal circulation of 6 mL/min. In the intervillous space at the maternal site of the cotyledon 4 cannulas were inserted establishing a maternal circulation of 12 mL/min. These flows are within the range commonly used within the field and considered physiological (van Hove, 2022). After a pre-perfusion period of 30–45 min, in which the system was stabilized and remaining blood was flushed out of the cotyledon, the pre-perfusion buffers were switched for clean experimental buffers and both the maternal and fetal circuit were closed to achieve recirculation. Antipyrine (100 µg/mL) and tofacitinib (100 nM) were added to the maternal reservoir (200 mL), whereas FITC dextran (50 µg/mL) was added to the fetal reservoir (200 mL). The cotyledons were perfused for 180 min during which 10 samples (t = 0, 1, 5, 15, 30, 60, 90, 120, 150, 180 min) were taken from both the fetal and maternal circulations. At the sampling times, the remaining volume of both perfusion buffers was estimated to inspect if volume loss from the fetal circulation occurred, other than due to the sampling procedure (i.e.. volume loss due to transfer of volume from the fetal circulation to the maternal circulation). After collections, samples were centrifuged for 5 min at 5000g and supernatant was stored at −20 °C for subsequent analysis of antipyrine, FITC-dextran and tofacitinib. To exclude that drug disappearance from the perfusion medium was due to adherence to perfusion system components or compound instability, experiments in absence of a placental cotyledon were performed before conducting the actual placenta perfusion experiments. In these studies, the concentration of tofacitinib in the maternal buffer did not decrease during 180 min of circulating the buffer through the system (see Supplementary Table S3). This excluded any relevant system adherence or instability of the compound under the test conditions.

### Quality criteria

Given the variety of ways *ex vivo* human cotyledon perfusions are conducted, a combined effort of European groups is currently underway to harmonize protocols. This includes recommendations on the use of quality criteria for successful perfusions (Schneider, 2022). While such criteria and corresponding cut-off values are not harmonized yet, most groups include loss of fluid from the fetal circulation, and maternal-to-fetal transfer of the small molecule antipyrine (van Hove, 2022). Fluid loss helps to evaluate integrity of the cotyledon as such and antipyrine transfer is used to evaluate if there is overlap of fetal and maternal circulations (i.e. successful cannulation). In the current set of experiments, we additionally included fetal-to-maternal transfer of the high molecular weight compound FITC-dextran, to specifically address the integrity of the fetal capillary bed.

For the placenta perfusions performed here (i.e. closed maternal circuit/closed fetal circuit, maternal circuit flow 12 mL/min, fetal circuit flow 6 mL/min, duration of perfusion 180 min) we consider experiments successful when fluid loss from the fetal circuit is less than 3 mL/h, that the fetal-to-maternal transfer of the high molecular weight compound FITC-dextran is low (ratio approximately 0.01) and the maternal-to-fetal antipyrine transfer ratio is high (>0.8). At the flow rates we operate, antipyrine concentrations are usually equal in both circuits after approximately 120 min of perfusion) (Eliesen, 2017, Eliesen, 2020, Freriksen, 2018, Schakenraad, 2021)*.*

### FITC-dextran and antipyrine assays

FITC-labeled dextran is a large molecule which cannot diffuse passively through the placenta. When added to the fetal perfusion solution it can be used to test the integrity of the capillary bed of the cotyledon. If FITC-dextran does not leak from the fetal vessels into the maternal reservoir, the capillaries remained intact during the experiment. The amount of FITC-dextran in the perfusion solutions was determined by measuring its fluorescence with a Multi-well Plate Reader (Perkin-Elmer®, excitation/emission: 485/530 nm).

Antipyrine is a small molecule that rapidly diffuses across the placental barrier and is hence often used as a quality control marker for placenta perfusion experiments. Antipyrine measurements in both perfusion solutions are used to visualize overlap of the maternal and fetal circulations. For antipyrine measurements, perfusion samples were deproteinized with 6 % perchloric acid. Subsequently, a reaction mix consisting of 0.2 mg/mL NaNO_2_ and 0.6 % concentrated H_2_SO_4_, was added to the perfusion samples (1:1). Formed nitro-antipyrine was determined via absorbance measurements at 350 nm, using a Biorad® Multi-well Plate reader.

### Tofacitinib measurements

The concentration of tofacitinib in the perfusion buffers was measured via LC-MS/MS. For the calibration curve (concentration range between 0 and 200 nM), the stock solution was diluted in 75 % MeOH and 10 µl of this diluted standard was added to 40 µl of perfusion buffer. Acetonitrile (150 µl) with 1 % formic acid was added to both calibration curve samples and 50 µl perfusion samples. After vortexing and centrifuging, the supernatant was transferred into vial and 1 µl was injected into the LC-MS/MS system that consisted of an Acquity UPLC (Waters, Milford, MA, USA) coupled to a Xevo TQ-S micro (Waters) triple quadrupole mass spectrometer. The tofacitinib was separated using an Acquity UPLC BEH column (Waters, 2.1 x 50 mm, 1.7 µm. The mobile phase consisted of solvent A (0.1 % formic acid in water) and solvent B (0.1 % formic acid in MeOH). Separation was achieved at a flow rate of 300 µl/min under the following gradient conditions: 0 min 95 % eluent A, 2.5 min 50 % eluent A, 4 min 0 % eluent A, 5 min 95 % eluent A. The effluent from the UPLC was passed directly into the electrospray ion source. Positive electrospray ionization was achieved using nitrogen as a desolvation gas with ionization voltage at 2000 Volt. The source temperature was set at 500 °C and argon was used as collision gas. The following SRM transitions were used: *m*/*z* 313.10 (parent ion) to *m*/*z* 149.01 and *m*/*z* 97.90 (product ions).

### Data analyses

Data analyses and construction of graphs was done using Graphpad Prism version 9 and version 10 for macOS. Data are presented as arithmetic mean ± SD.

## Results

**Fig. 1:**
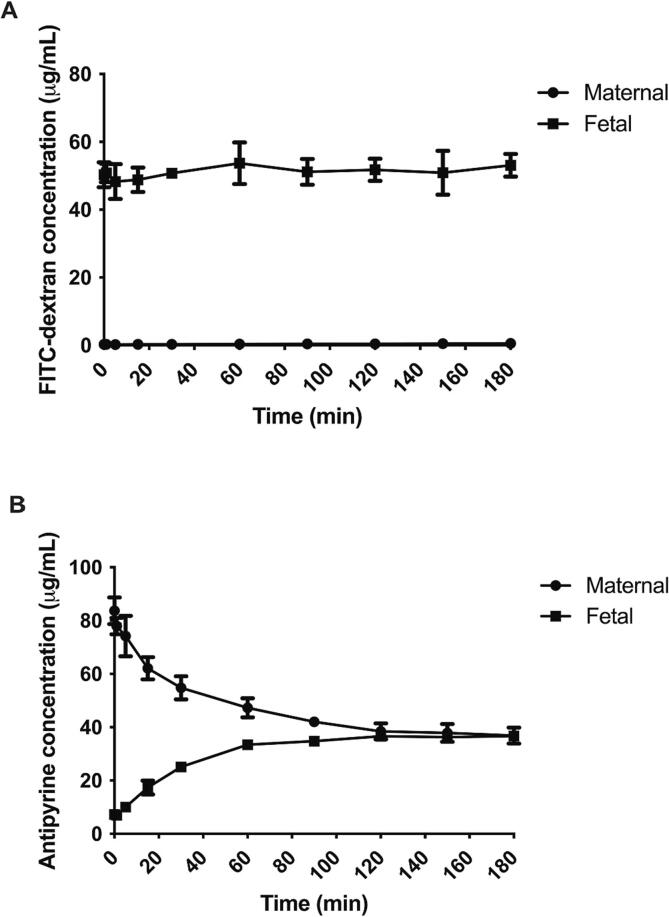
Course of the vascular integrity marker FITC-dextran (A) and passive diffusion marker Antipyrine (B) concentrations over time in placenta perfusions. Data are expressed as mean ± SD. Data are from n = 3 cotyledon perfusions from 3 independent placenta donors.

Three successful placenta perfusions with tofacitinib were performed in cotyledons obtained from different donors. In all experiments volume leakage from the fetal to maternal buffer was minimal (range 0–1.7 mL/h at the end of the perfusions). In Fig. 1A it can be seen that FITC-dextran did not traverse the placental barrier. After adding a concentration of 50 µg/mL to the fetal circuit, the concentration in the fetal buffer remained stable. No relevant FITC-dextran ended up in the maternal circulation (for all perfusion this was < 1 µg/mL). The data from the individual experiments can be found in Supplementary Table S1. Degree of fetal-to-maternal transfer, expressed as the ratio of maternal concentration divided by the fetal concentration at the end of the perfusions, was 0.01 ± 0.01. This indicates that in all experiments fetal capillaries were intact. Recovery of FITC dextran from the combined perfusates was 107 ± 7 % of the amount initially added, indicating no relevant system adherence or tissue accumulation. The latter further underlines vascular integrity.

In contrast, the small molecule diffusion marker antipyrine rapidly transferred across the placental barrier, reaching an equilibrium between the maternal and fetal circulation from 120 min of perfusion onwards (Fig. 1B). Final antipyrine maternal and fetal concentrations amounted to 36.9 ± 3.0 and 36.7 ± 1.3 µg/mL, respectively (ratio of maternal-to-fetal transfer was 1.00 ± 0.05). At the end of the perfusion period of 180 min, 74 ± 4 % of the added antipyrine was recovered from the combined maternal and fetal perfusates. The data from the individual experiments can be found in Supplementary Table S2. The rapid and complete equilibration of antipyrine concentrations between both circulations demonstrates good overlap between the maternal and fetal circuit and hence confirms correct cannulation of the cotyledon.

**Fig. 2:**
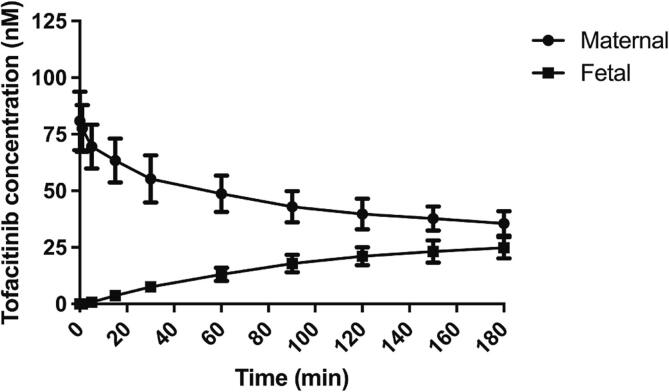
Tofacitinib concentration over time in placenta perfusions. Data represent mean ± SD from n = 3 cotyledon perfusions from 3 independent placenta donors.

Fig. 2 displays the placental handling of tofacitinib. It can be clearly seen that tofacitinib rapidly and extensively crosses the placenta. After 180 min. of perfusion, equilibrium is already almost reached, which is similar as observed for the passive diffusion marker antipyrine. At the end of the perfusion period, drug concentrations in the maternal and fetal reservoirs were near equilibrium, at 35.6 ± 5.5 and 24.8 ± 4.7 nM, respectively. 61 ± 10 % of the added tofacitinib was recovered from the combined maternal and fetal perfusates at the end of the perfusion period of 180 min. Tofacitinib exhibited no relevant system adherence (see Supplementary Table S3). The recovery percentage obtained from the perfusates may therefore point to tissue accumulation or, possibly, biotransformation taking place. The data from the individual experiments can also be found in Supplementary Table S3.

## Discussion

Maternal exposure to tofacitinib during the first trimester has been reported in women who became pregnant unexpectedly while on tofacitinib treatment. To date, no intentional use of tofacitinib during pregnancy has been reported. Therefore, data on safety as well as placental transfer and fetal exposure are lacking. In this study, we showed that tofacitinib rapidly crosses the placental barrier in an *ex vivo* experimental set-up.

It is expected that the current *ex vivo* findings are a good indicator of the clinical placental pharmacokinetics of tofacitinib in 3rd trimester. Dual-side *ex vivo* placenta perfusion is the gold standard technique to study placental drug disposition. Generally, the technique provides a good match between *ex vivo* and *in vivo* observations (van Hove, 2022). It should be noted that in specific cases, mismatch between *ex vivo* and *in vivo* observations occurs. This often results from not taking into account differences in protein binding between maternal and fetal perfusate/plasma or when the proper binding proteins are not added to the perfusion media (Hutson, 2011). Other sources of discrepancy are a very slow transfer rate of the pharmaceutical under investigation. In our lab, placenta perfusions can be maintained for 3–6 h after which vascular integrity is no longer adequate. It is thus difficult to establish full extent and rate of transfer in the *ex vivo* set-up when transfer of a molecule occurs much slower. Discrepancies between *ex vivo* and *in vivo* placental transfer may also occur when compounds have a high degree of partitioning into erythrocytes, as these are not present in the perfusion medium (Freriksen, 2018). However, tofacitinib appears to be a molecule for which placental transfer can be readily established *ex vivo*. Firstly, tofacitinib exhibits protein binding to albumin. We included albumin at physiological levels in the perfusion buffers, hence mimicking the *in vivo* situation (European Medicines Agency, 2017). Furthermore, placental transfer of tofacitinib could be readily investigated within the experimental duration (European Medicines Agency, 2017). Finally, tofacitinib does not accumulate extensively in erythrocytes, i.e. the absence of erythrocytes in the perfusion set-up should also not perturb *ex vivo-in vivo* extrapolation of the results. It should be noted that we studied placentas from healthy pregnancies. It could be that placental barrier function is different in placentas from women with autoimmune disorders. To our knowledge this has not been charted and should be topic of future study.

Although extensive clinical safety data are lacking, in preclinical studies tofacitinib was found to be teratogenic. Visceral and skeletal abnormalities were found in rats at doses of over 100 mg/kg/day in presence of maternal toxicity, and in rabbits in absence of maternal toxicity at doses of at least 30 mg/kg/day. These respectively correspond to 58- and 2.9- times the clinical exposure level (total AUC_0-24h_ in humans at a dose 5 mg twice daily) and should be considered relevant to the clinical situation (European Medicines Agency, 2017, European Medicines Agency, 2017). Especially maternal exposure levels in rabbits do not deviate much from those observed in humans. Furthermore, effects on female fertility were observed at, such as reduced number of liveborn pups and reduced pup survival at 50 mg/kg/day and a decrease in implantation sites and increase in early resorptions at 100 mg/kg/day (European Medicines Agency, 2017, European Medicines Agency, 2017).

Tofacitinib is an inhibitor of all JAK-proteins. JAK/STAT signaling is essential for many developmental and homeostatic processes. For example, hematopoiesis, immune cell development, stem cell maintenance, and mammary gland development (Ghoreschi et al., 2009). Placental *in vitro* studies suggest that placental trophoblasts are largely dependent on JAK/STAT signaling. Interference with this can affect cell proliferation and invasiveness, two crucial processes for adequate placentation (Pereira de Sousa, 2017). Although much is still unknown on the role of the JAK-STAT pathway in embryonal, fetal and placental development, the consequences of inhibition by tofacitinib could be serious given the role of the signaling pathway in various basic cell processes. Not only during organogenesis, which takes place in the early stages of pregnancy, but during the whole course of gestation.

Despite clear adverse effects observed in preclinical animal species, drugs may still be employed during pregnancy, for example in case of a pressing medical need. A good example of which is the treatment of pregnant women with cancer, for which the classic cytostatic agents are an accepted treatment option. By virtue of their mechanism of action, virtually all of these have intrinsic teratogenic effects. Nevertheless, in the later stages of pregnancy their use can be considered, particularly in the absence of better alternatives (Maggen, 2022, Berveiller, 2016).

Given the wide range of JAK pathways that tofacitinib exhibits, its potential to inhibit angiogenesis, in combination with the clear preclinical reproductive toxicological findings that have been reported, prescribing tofacitinib during pregnancy does not appear to be an obvious choice (Di Benedetto, 2021, European Medicines Agency, 2017, Gotestam Skorpen, 2016). Moreover, at the moment, a relatively large body of clinical data is available on the use biological DMARDs for treatment of auto-immune diseases in pregnant patients. This is particularly the case for the TNF-alpha inhibitors, which have been on the market for quite some time, such as infliximab or adalimumab. Despite extensive placental transfer of many of these drugs, as a result of FcRn-mediated placental transfer, the biologicals are considered to be safe during pregnancy as pregnancy outcomes are generally good (Narula, 2014). However, not all patients respond well to TNF-alpha inhibitors. For example, up to 40 % of rheumatoid arthritis patients do not respond to the first biologic (primary failure) or lose response over time (secondary failure). An important reason for secondary failure is the production of antibodies that are targeted against the anti-TNF biologicals (Fernandez, 2023, Mehta and Manson, 2020). This illustrates that investigating the safety of novel drugs remains important for specific difficult-to-treat pregnant subpopulations. The same holds true for novel therapies that highly improve disease outcome compared to the more conventional treatments.

Currently, cohort studies are ongoing to study the safety of drugs for treatment of autoimmune diseases such as the DUMBO study where the focus is on patients with inflammatory bowel disease (Chaparro, 2021). This will extend our knowledge on the potential use of the more novel biologics as well as novel small molecules, as up to now, we mostly rely on data from case reports and case series.

In summary, in the *ex vivo* perfused term placenta tofacitinib traverses the placental barrier rapidly and extensively. This suggests that substantial fetal tofacitinib exposure will take place after maternal drug dosing towards the end of gestation. Transfer at earlier gestational age remains to be investigated in more detail. Should tofacitinib be used to treat pregnant women, substantial fetal exposure is likely and should be taken into consideration by the treating physician.

## Funding

Gaby A.M. Eliesen was supported by a personal grant from Radboud university medical center.

## CRediT authorship contribution statement

**Gaby A.M. Eliesen:** Conceptualization, Methodology, Investigation. **Milou Fransen:** Investigation. **Hedwig van Hove:** Methodology, Investigation. **Petra H.H. van den Broek:** Methodology, Investigation. **Rick Greupink:** Conceptualization, Methodology, Investigation.

## Declaration of competing interest

The authors declare that they have no known competing financial interests or personal relationships that could have appeared to influence the work reported in this paper.

## Data Availability

Data will be made available on request.
