# Supplementary material for: Placental transfer of tofacitinib in the ex vivo dual-side human placenta perfusion model
Source: Curr Res Toxicol. 2024 Jan 8;6:100149. doi: 10.1016/j.crtox.2024.100149 (PMC10825226; doi:10.1016/j.crtox.2024.100149)
Supplement: Supplementary data 1 [file mmc1.docx]

**SUPPLEMENTARY FILE TO:**

**Placental transfer of tofacitinib in the *ex vivo* dual-side human placenta perfusion model**

Gaby A.M. Eliesen^1,2^, Milou Fransen^1^, Hedwig van Hove^1^, Petra H.H. van den Broek^1^, Rick Greupink^1,3^

*^1^ Department of Pharmacology and Toxicology, Radboud university medical center, Nijmegen, The Netherlands.*

*^2^ Centre for Safety of Substances and Products, National Institute for Public Health and the Environment, Bilthoven, the Netherlands.*

*^3^ Author to whom correspondence should be addressed.*

**Table S1.** Perfusate FITC-dextran concentrations of the individual experiments.

|  | FITC-dextran fetal (µg/ml) | | | FITC-dextran maternal (µg/ml) | | |
| --- | --- | --- | --- | --- | --- | --- |
| Time (min) | Placenta 1 | Placenta 2 | Placenta 3 | Placenta 1 | Placenta 2 | Placenta 3 |
| 0 | 54.5 | 48.5 | 47.9 | 0.6 | 0.3 | < 0.1 |
| 1 | 51.7 | 53.0 | 47.8 | 0.6 | 0.3 | < 0.1 |
| 5 | 53.4 | 43.2 | 48.4 | 0.6 | 0.3 | < 0.1 |
| 15 | 52.4 | 45.2 | 48.9 | 0.6 | 0.3 | < 0.1 |
| 30 | 51.9 | 50.3 | 50.1 | 0.6 | 0.4 | < 0.1 |
| 60 | 52.7 | 60.3 | 48.1 | 0.7 | 0.4 | < 0.1 |
| 90 | 51.0 | 55.0 | 47.4 | 0.7 | 0.5 | < 0.1 |
| 120 | 52.0 | 54.9 | 48.3 | 0.8 | 0.6 | < 0.1 |
| 150 | 50.8 | 57.4 | 44.5 | 0.8 | 0.7 | < 0.1 |
| 180 | 50.2 | 56.8 | 52.3 | 0.8 | 0.9 | < 0.1 |

**Table S2.** Perfusate antipyrine concentrations individual experiments.

|  | Antipyrine maternal (µg/ml) | | | Antipyrine fetal (µg/ml) | | |
| --- | --- | --- | --- | --- | --- | --- |
| Time (min) | Placenta 1 | Placenta 2 | Placenta 3 | Placenta 1 | Placenta 2 | Placenta 3 |
| 0 | 83.2 | 88.9 | 79 | 8.0 | 6.4 | 7.3 |
| 1 | 80.4 | 79.1 | 74.5 | 8.6 | 5.8 | 6.5 |
| 5 | 81.3 | 75.2 | 66.2 | 11.3 | 10.0 | 8.8 |
| 15 | 64.6 | 64.4 | 57.3 | 17.5 | 19.9 | 14.6 |
| 30 | 57.3 | 57.2 | 49.8 | 24.3 | 26.8 | 24.3 |
| 60 | 50.7 | 47.7 | 43.5 | 32.9 | 33.9 | 33.7 |
| 90 | 42.7 | 41.8 | 41.7 | 35.9 | 34.9 | 33.5 |
| 120 | 41.1 | 39.1 | 35.1 | 36.4 | 37.1 | 36.5 |
| 150 | 38.8 | 40.7 | 34.2 | 37.0 | 37.7 | 34.2 |
| 180 | 39.6 | 37.4 | 33.7 | 38.0 | 36.8 | 35.4 |

**Table S3.** Perfusate tofacitinib concentrations for the individual placenta perfusions, as well as from a control perfusion (without placenta) to assess system adherence.

|  | Tofacitinib control (nM) | Tofacitinib maternal (nM) | | | Tofacitinib fetal (nM) | | |
| --- | --- | --- | --- | --- | --- | --- | --- |
| Time (min) | No placenta in system | Placenta 1 | Placenta 2 | Placenta 3 | Placenta 1 | Placenta 2 | Placenta 3 |
| 0 | 100.9 | 95.8 | 75.2 | 71.8 | 0 | 0 | 0 |
| 1 | N/A | 89.4 | 70.5 | 72.6 | 0 | 0 | 0 |
| 5 | 105.9 | 80.7 | 64.5 | 63.4 | 1.8 | 0.3 | 0.3 |
| 15 | 99.7 | 74.1 | 55.4 | 60.6 | 4.9 | 3.1 | 3.2 |
| 30 | 98.6 | 67.4 | 48.7 | 49.8 | 9.6 | 6.1 | 6.9 |
| 60 | 100.7 | 57.9 | 43.8 | 44.4 | 16.1 | 10.1 | 13.2 |
| 90 | 97.0 | 50.9 | 38.7 | 39.3 | 21.8 | 13.9 | 18.0 |
| 120 | 105.7 | 47.7 | 35.9 | 35.7 | 25.1 | 17.2 | 21.2 |
| 150 | 106.4 | 43.9 | 35.1 | 34.3 | 28.0 | 18.0 | 23.7 |
| 180 | 122.8 | 41.9 | 33.5 | 31.5 | 30.0 | 20.1 | 24.9 |

N/A: not assessed.
